# Genetic Population Structure of the Italian Wall Lizards *Podarcis siculus* (Squamata: Lacertidae): Insight From Nuclear DNA Markers

**DOI:** 10.1002/ece3.72655

**Published:** 2026-01-15

**Authors:** Gabriele Senczuk, Chiara Ripa, Paolo Colangelo, Riccardo Castiglia

**Affiliations:** ^1^ Dipartimento di Agricoltura, Ambiente e Alimenti Università del Molise Campobasso Italy; ^2^ National Research Council Institute of Polar Sciences, Ca' Foscari University Venice Venezia Mestre Italy; ^3^ National Research Council Research Institute on Terrestrial Ecosystems Rome Italy; ^4^ Dipartimento di Biologia e Biotecnologie “Charles Darwin” Università degli studi di Roma “La Sapienza” Rome Italy

**Keywords:** Italian peninsula, Lacertidae, mediterranean lizards, phylogeography, zoogeography

## Abstract

Since the Quaternary, recurrent climatic oscillations have profoundly shaped species distributions across the Mediterranean basin, generating complex phylogeographic patterns through repeated cycles of range contraction and expansion. The Italian Peninsula, characterized by a highly heterogeneous topography and a mosaic of glacial refugia, has emerged as a hotspot of intraspecific genetic diversity. The Italian wall lizard (*Podarcis siculus*), a widespread species across the Italian Peninsula and adjacent islands, represents an excellent model for exploring these dynamics. Previous phylogeographic studies based on mitochondrial (mtDNA) and nuclear (nuDNA) markers have revealed a highly structured matrilineal organization, with several parapatric lineages and subclades, but have also highlighted weak differentiation in nuDNA, suggesting possible mito‐nuclear discordance. In this study, we used multilocus nuclear microsatellite data to (i) evaluate whether the complex mtDNA phylogeographic structure is reflected in nuclear markers, or whether evidence of mito‐nuclear discordance is present, and (ii) quantify admixture and gene flow among mitochondrial lineages. Our results reveal partial congruence between mtDNA and nuclear patterns, with evidence of both historical isolation and secondary contact among major clades. However, several populations exhibit substantial mito‐nuclear mismatches, suggesting a history of asymmetric introgression and differential lineage sorting. These findings indicate that *P. siculus* experienced multiple phases of demographic expansion and secondary contact, consistent with Pleistocene‐driven range dynamics, and that mito‐nuclear discordance has played a significant role in shaping its current genetic landscape. This study underscores the importance of integrating multilocus nuclear data with mitochondrial evidence to disentangle the evolutionary processes driving complex phylogeographic patterns in Mediterranean taxa.

## Graphical abstract

Microsatellite analysis of 294 *Podarcis siculus* specimens from 121 localities revealed eight genetic clusters that partially align with previously identified mtDNA lineages. However, notable mito‐nuclear discordance was observe, particularly in Southern Italy and Sicily, where microsatellite data indicated different contact zones and less genetic structure than mtDNA suggested. These discrepancies likely stem from factors including differential introgression, distinct dispersal patterns (e.g., sex‐biased philopatry), and varying evolutionary timescales captured by mitochondrial versus nuclear markers.

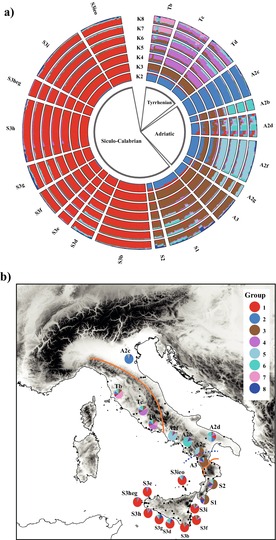

## 1 Introduction

Since the Quaternary period, cyclic climatic fluctuations have profoundly influenced both environmental landscapes and species distributions, driving diverse biogeographical responses. In recent decades, the extensive use of genetic data, leveraging both mitochondrial and nuclear markers, has facilitated the development of general models of range expansion and contraction to explain the genetic distribution patterns currently observed in numerous Mediterranean taxa (Taberlet et al. 1998; Hewitt 2000; Habel et al. 2005; Schmitt 2007; Ahmadi et al. 2021; Domínguez et al. 2023). The complexity of these processes encompasses classical scenarios of population contraction towards more climatically suitable southern refugia during glacial periods, followed by interglacial northward expansions. Additionally, contrasting patterns have been documented, particularly in Southern Mediterranean regions, where marine regressions during glacial episodes expanded climatically favorable coastal lowlands, thereby promoting population persistence and expansions (Bisconti et al. 2011; Salvi et al. 2014; Senczuk et al. 2019). This heterogeneity in both species responses and environmental dynamics has created favorable conditions for the emergence and persistence of exceptional biodiversity within the three major Mediterranean peninsulas (Taberlet et al. 1998; Weiss and Ferrand 2007). Among these, the Italian Peninsula stands out as a hotspot of genetic diversity, where its complex orography, characterized by extensive mountain ranges, has facilitated the formation of multiple glacial refugia (Schmitt et al. 2021; Chiocchio, Maiorano, et al. 2024).

The Italian wall lizard, *Podarcis siculus*, is sub‐endemic to the Italian Peninsula, as well as the islands of Sicily, Sardinia, and Corsica, and extends along the Croatian coast and numerous smaller islands and islets. This species represents a model organism for phylogeographic studies, having been the subject of numerous investigations over the past decades. These studies have primarily focused on the role of Pleistocene paleoclimatic contexts and insular conditions in shaping genetic variability (Podnar et al. 2005; Senczuk et al. 2017; Senczuk, Havenstein, et al. 2018; Senczuk, Colangelo, et al. 2018; Sabolić et al. 2024; Sherpa et al. 2024). Furthermore, many studies have concentrated on reintroduction sites, as the species is highly adaptable and has been widely translocated beyond its native range by human activities (Oskyrko et al. 2022; Patti et al. 2023). Specifically, phylogenetic studies based on both mitochondrial (mtDNA) and nuclear (nuDNA) markers have revealed a complex evolutionary history shaped by multiple processes, including allopatric divergence within several southern refugia and more recent range expansions, likely occurring during the Last Glacial phase (Podnar et al. 2005; Senczuk et al. 2017; Senczuk, Havenstein, et al. 2018; Senczuk, Colangelo, et al. 2018).

Notably, the matrilineal structure of *P. siculus* reveals a geographically nested diversity of lineages. It includes two primary parapatric lineages: the Siculo‐Calabrian (S) lineage and the Central‐Northern (CN) lineage, with their contact zone located in central Calabria. Furthermore, the CN lineage is subdivided into two additional parapatric clades: the Tyrrhenian (T) and Adriatic (A) clades. Similarly, the S lineage is further divided into three clades: S1, S2, and S3. Moreover, almost every clade displays a distinct internal structure, characterized by multiple haplogroups, reflecting a complex phylogeographic history.

Despite the intricate phylogeographic structure revealed by mtDNA, nuDNA has proven to be less informative in distinguishing mtDNA lineages within *P. siculus* . However, some degree of differentiation was observed when analyzing nuDNA haplotype frequencies across several mtDNA haplogroups (Senczuk et al. 2017; Senczuk, Havenstein, et al. 2018; Senczuk, Colangelo, et al. 2018). More recently, a low‐density SNP panel has been used on specific island systems to reconstruct the colonization history (Sherpa et al. 2024); however, a comprehensive study covering the species' entire range is still lacking.

Mito‐nuclear discordance is a widespread phenomenon observed across many taxa (Toews and Brelsford 2012), and it appears to be a common pattern along the Italian Peninsula (e.g., Canestrelli et al. 2007; Bisconti et al. 2018; Chiocchio, de Rysky, et al. 2024). Although multiple mechanisms have been implicated—including incomplete lineage sorting, introgression, sex‐biased dispersal, and selection on mtDNA—the relative contributions of these processes remain difficult to disentangle (Bonnet et al. 2017; Bailey and Stevison 2021). Several key issues continue to challenge our understanding, including the extent to which selective versus demographic processes drive discordance, the functional significance of mitonuclear mismatches, the role of sex‐biased gene flow, and the potential for such discordance to contribute to reproductive isolation (Bonnet et al. 2017; Burton 2022; Chiocchio, de Rysky, et al. 2024). The multitude of factors underlying mitonuclear discordance necessitates the analysis of numerous case studies. *Podarcis siculus*, with its wide distribution across the Italian Peninsula, a region shaped by a complex paleogeographic history, and high mtDNA lineages with parapatric distribution, represents an ideal model for investigating this phenomenon.

In this paper, we employed microsatellite loci to: (i) assess whether the complex and nested genetic substructure detected in mtDNA is mirrored in nuclear microsatellite markers and therefore evaluate potential instances of mito‐nuclear discordance, and (ii) determine the extent of admixture resulting from gene flow between mtDNA haplogroups.

## 2 Materials and Methods

### 2.1 Samples Collection and Genotyping

**FIGURE 1:**
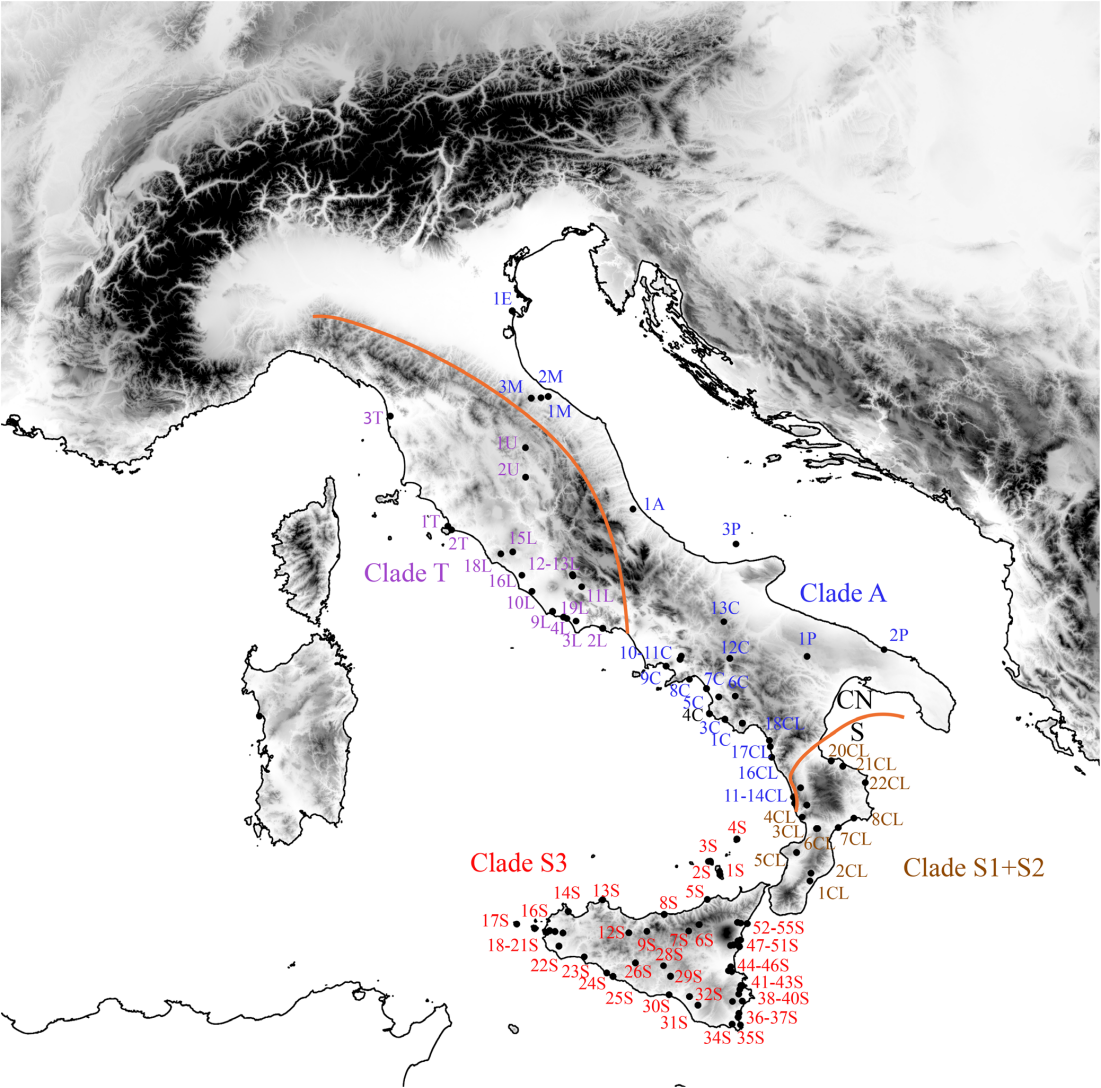
Map of the study area displaying the sampled locations of *Podarcis siculus* analyzed in this study (see Table S2 for details). The genetic discontinuities between the two lineages CN and S, as well as among the main clades are reported with orange lines. Different colors indicate membership in distinct clades within the main lineages: Clades S1, S2, and S3 within the S lineage, and clades A and T within the CN lineage.

A total of 293 *Podarcis siculus* specimens from 121 localities were analyzed using microsatellite markers (see Figure 1 and Table S1 for details). These specimens correspond to the same individuals previously examined in earlier studies (Senczuk et al. 2017; Senczuk, Havenstein, et al. 2018; Senczuk, Colangelo, et al. 2018). Genomic DNA was extracted from tail muscle tissue preserved in 96% ethanol, following the universal protocol of Salah and Martinez (1997), which includes incubation at 56°C with proteinase K and DNA precipitation with isopropanol.

All samples were genotyped using a panel of 11 microsatellite loci previously published: C9 (Nembrini and Oppliger 2003), Pb10, Pb73 (Pinho et al. 2004), Pli3, Pli4, Pli10, Pli18, Pli24, Pli21 (Bloor et al. 2011), and Lv19, Lv4a (Boudjemadi et al. 1999) (see Tables S2 and S3 for primer sequences and PCR conditions). These loci have been shown to be polymorphic in different *Podarcis* species (Runemark et al. 2008) including in *P. siculus* (Gallozzi et al. 2023).

Multiplex PCR amplifications were performed for two sets of loci (Pli10‐Pb73 and Pli4‐Pli24) and subsequently compared with singleplex PCR to verify the absence of allele dropouts and inconsistencies in allele sizing. PCR products were purified and genotyped at Eurofins Genomics. Allele sizes were determined using PEAK SCANNER v.1.0 software (Applied Biosystems) by comparison with an internal size standard (LIZ500).

### 2.2 Data Analysis

To enhance the estimation of gene flow and the interpretation of admixture patterns among populations while minimizing potential biases from low sample sizes in certain locations, the dataset was organized into 20 groups according to the parapatric mtDNA haplogroups identified by Senczuk et al. (2017). Each haplogroup is labeled with an initial letter corresponding to its respective mitochondrial clade.

The average allelic richness (Ar), the expected (*H*_e_) and observed (*H*_o_) heterozygosity, the inbreeding coefficient (*F*_IS_), null alleles, and deviation from Hardy–Weinberg were calculated for each haplogroup using the R package Hierfstat v0.5‐11 (Goudet 2005). Since we identified suspected null alleles, to assess the potential impact on the overall genetic diversity and population structure, some analyses have been analyzed excluding those loci (see Section 3).

To analyze the distribution of genetic variation while accounting for spatial autocorrelation, a spatial Principal Component Analysis (sPCA) was conducted using the R package Adegenet (Jombart 2008; Jombart and Ahmed 2011). Unlike conventional PCA, this multivariate approach maximizes the product of genetic variance and spatial autocorrelation, thereby enabling a more effective distinction between global and local genetic structure. Importantly, this analysis does not require the data to conform to Hardy–Weinberg expectations or to linkage equilibrium among loci (Jombart et al. 2008).

To further investigate genetic structure, we employed a Bayesian clustering algorithm as implemented in the R package tess3r (Caye and Francois 2016). One advantage of TESS3 is that, similarly to sPCA, it is not sensitive to deviations from Hardy–Weinberg equilibrium (Caye et al. 2016), thereby allowing reliable estimation of admixture proportions even in the presence of such deviations.

The analysis was performed using *K* values ranging from 1 to 20, with five replicates for each *K* value. The resulting *Q*‐matrix was used to visualize in a circular fashion using the membercoef.circos function in the R package BITE (Milanesi et al. 2017), while the average genetic component of each population was plotted at sampling locations through pie charts. The most likely genetic clusters were determined based on the Bayesian Information Criterion (BIC) and Δ*K* (Evanno et al. 2005).

To investigate the possible occurrence of isolation by distance (IBD), we performed a Mantel test between the *F*_ST_ matrix, calculated among the 20 groups, and their geographic distances using the function mantel.randtest from the package Ade4 (Dray and Dufour 2007).

Finally, to assess the extent of gene flow among the different mtDNA haplogroups, we utilized the R package diveRsity (Keenan et al. 2013). The function divMigrate implements the method described by Sundqvist et al. (2016) to estimate relative migration levels between haplogroups. Specifically, we employed Jost's *D* distances to generate a complete migration matrix for the 20 haplogroups, which were then visualized using a heatmap.

## 3 Results

### 3.1 Genetic Diversity

Estimates of null allele frequencies and deviations from Hardy–Weinberg equilibrium are reported in Tables S4 and S5. Two loci (L9 and L10) exhibited consistently high null allele frequencies (> 0.20) across populations, suggesting potential technical artifacts rather than genuine population‐level processes. To minimize bias in summary statistics, these loci were excluded from estimates of genetic diversity (e.g., heterozygosity, allelic richness, and *F*_IS_). However, they were retained in the clustering analyses, as both TESS3 and sPCA are robust to deviations from Hardy–Weinberg equilibrium. Indeed, excluding the two loci did not affect the inferred population structure. Therefore, results on population structure (admixture proportions and spatial components) are reported based on the complete dataset including all 11 loci.

Expected and observed heterozygosity (*H*_e_, *H*_o_), allelic richness, and inbreeding coefficient (*F*_IS_) across 9 loci for each of the 20 haplogroups is presented in Table 1.

**TABLE 1:** Sample size (*N*), average allelic richness (Ar), expected (*H*_e_), observed (*H*_o_) heterozygosity, and inbreeding coefficient (*F*_IS_) for each haplogroup. The assignment of haplogroups to the main mtDNA lineages and clades, defined according to Senczuk et al. (2017), is also indicated.

mtDNA lineage	mtDNA Clade (Senczuk et al. 2017)	Haplogroup	*N*	Ar	*H*_e_	*H*_o_	*F*_IS_
S	S3	S3ieo	20	4.64	0.79	0.64	−0.19
S	S3	S3i	27	5.49	0.89	0.59	0.34
S	S3	S3g	12	4.37	0.73	0.55	0.22
S	S3	S3h	30	4.36	0.74	0.66	0.10
S	S3	S3e	6	3.30	0.59	0.59	0.00
S	S3	S3heg	8	3.76	0.69	0.61	0.15
S	S3	S3d	11	4.74	0.83	0.67	0.16
S	S3	S3b	27	5.01	0.84	0.62	0.24
S	S3	S3f	13	4.49	0.77	0.50	0.36
S	S1	S1	24	5.05	0.83	0.63	0.24
S	S2	S2	5	3.78	0.77	0.66	0.11
CN	A	A3	12	4.08	0.71	0.59	0.16
CN	A	A2g	6	3.44	0.65	0.67	−0.03
CN	A	A2d	11	4.84	0.83	0.63	0.26
CN	A	A2c	16	4.43	0.75	0.64	0.17
CN	A	A2f	20	5.15	0.85	0.69	0.20
CN	A	A2b	5	3.91	0.70	0.51	0.32
CN	T	Tc	16	5.32	0.85	0.63	0.26
CN	T	Td	14	4.59	0.77	0.63	0.29
CN	T	Tb	10	4.06	0.72	0.64	0.12

Expected heterozygosity across the 20 haplogroups ranged from 0.59 (S3e and A2g) to 0.89 (S3i), while observed heterozygosity ranged from 0.5 (S3f) to 0.69 (A2f). The inbreeding coefficients (*F*_IS_) are negative in only two groups (S3ieo and A2g), while all the other values are positive, with the highest value being 0.36 (S3f). These observed positive *F*_IS_ values indicate heterozygote deficiency and may be due to the fact that the individuals included in our groups originate, at least in part, from different populations. Overall, Sicilian haplogroups displayed lower genetic diversity indices compared to other Peninsular haplogroups (Table 1).

### 3.2 Population Structure and Gene Flow

**FIGURE 2:**
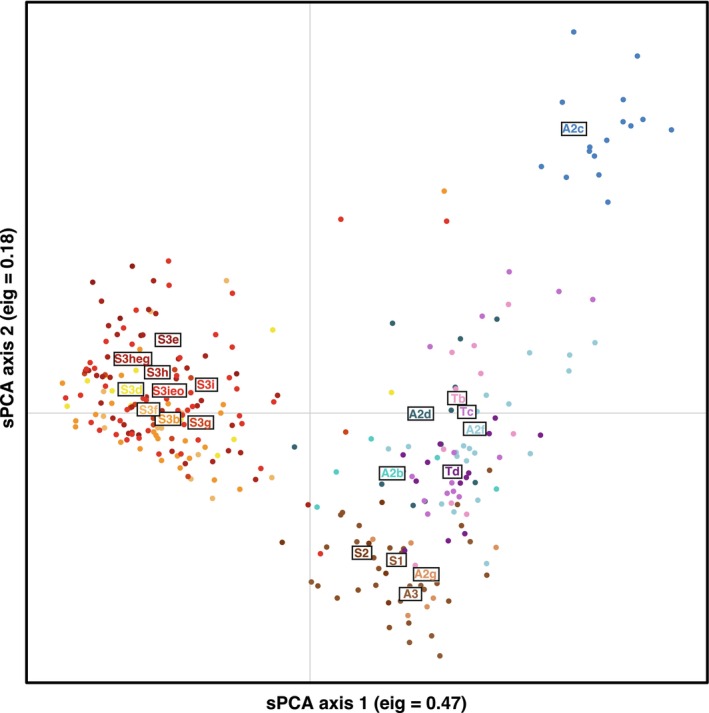
Results of the spatial Principal Component Analysis (sPCA) based on individual genotypes for 11 microsatellite loci. Dots represent individuals; different colors indicate assignment to distinct mtDNA haplogroups.

The results of the spatial Principal Component Analysis (sPCA) revealed a clear association between microsatellite variability, geography, and mtDNA haplogroups. Specifically, the first axis distinguished all Sicilian samples from the Peninsular ones (Figure 2). Conversely, the second axis identified a geographic gradient along a South–North axis, with the Northern haplogroup (A2c) exhibiting the highest degree of differentiation.

**FIGURE 3:**
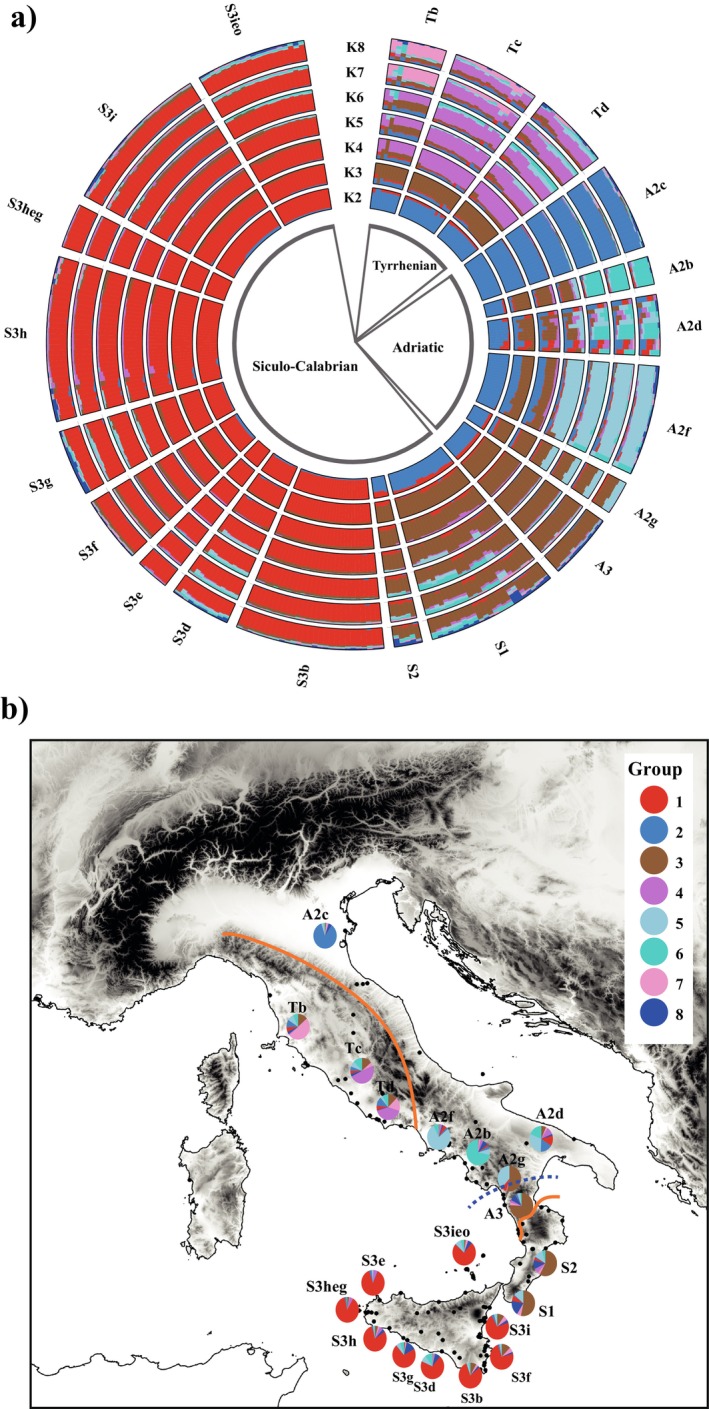
(a) Clustering analysis performed by tess3r on the entire dataset for *K* values ranging from 2 to 8. (b) Pie charts represent the average frequency distribution of the most likely number of genetic clusters (*K* = 8) across the 20 haplogroups. The boundary between the main lineages, CN and S, is indicated. The dotted line indicates a nuclear secondary contact zone located about 100‐km north of the CN/S main mitochondrial discontinuity (see text for explanation). Each haplogroup is designated by an initial letter corresponding to its respective mitochondrial clade (i.e., A and T for the CN lineage, and S1, S2, and S3 for the S lineage).

The clustering analyses performed by tess3r on the entire dataset (Figure 3a) identified *K* = 8 as the most likely number of genetic clusters according to the cross‐validation procedure (Figure S1). The earliest ancestral partition, detected at *K* = 2, revealed a separation between the Sicilian samples and all other insular and peninsular populations. At *K* = 3, a further differentiation emerged, with the A2c haplogroup, predominantly distributed in northeastern Italy, forming a distinct cluster. At *K* = 4, the Tyrrhenian lineage (Tb, Tc, and Td) clustered separately, while at higher *K* values, the progressive subdivision of additional haplogroups within the Adriatic lineage became increasingly evident. Although most mtDNA haplogroups correspond to specific nuclear microsatellite components, a certain degree of genetic admixture was observed in A2d and, to a lesser extent, in Tb (Figure 3). This geographic pattern of genetic structuring and admixture is further illustrated through pie charts displaying the average distribution of the components (Figure 3b).

We found no evidence of isolation by distance (IBD), as the Mantel test was not significant (*p* = 0.172; see Figure S2).

**FIGURE 4:**
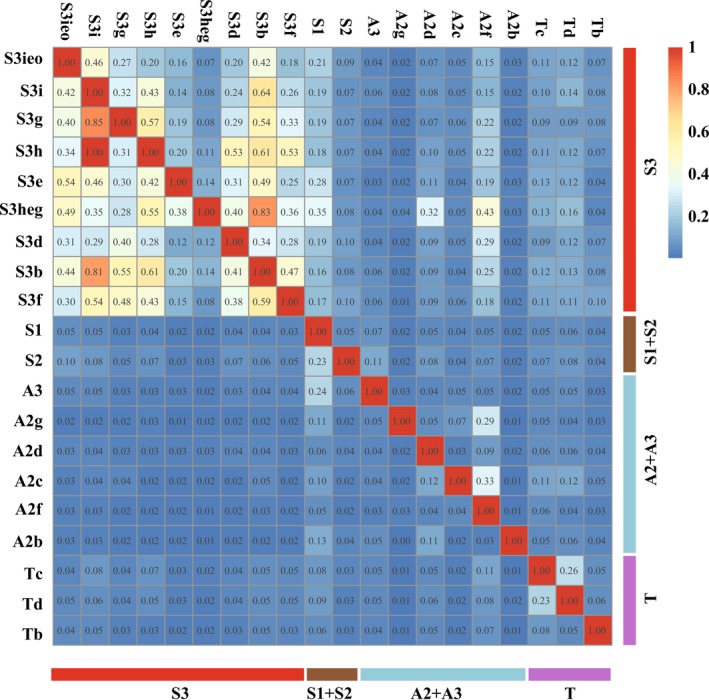
Heat map based on Jost's *D* distance matrix depicting pairwise relative migration levels between the 20 haplogroups. Each gene flow value corresponds to a specific source population (rows) and recipient population (columns). The belonging to different mitochondrial clades is also indicated. The legend displays the range of Jost's *D* distance values in relation to the color gradient (warm and cool tones) shown in the plot.

The gene flow analysis, visualized in the heatmap, largely corroborates the results of both the genetic structure analysis (Figure 4) and the Mantel test. Notably, gene flow appears particularly intense among all haplogroups of the Sicilian S3 clade, contrasting with the generally lower levels observed among peninsular groups. This pattern aligns with the absence of a general IBD signal in our dataset.

A lower yet detectable level of unidirectional gene flow was observed from the Sicilian groups towards the Calabrian haplogroup S1 and A2f, the latter located further north, as well as from S2 and A3 to S1, all of which are located in Calabria (Figure 4).

## 4 Discussion

The spatial distribution and frequency of the eight identified genetic clusters exhibit a geographic pattern that largely aligns with the mitochondrial (mtDNA) structure (Figure 3). However, several discrepancies are observed, primarily occurring in Southern Italy and Sicily.

Based on mtDNA, the deepest phylogenetic split separating the Siculo‐Calabrian (S) and Central‐Northern (CN) lineages (Senczuk et al. 2017) is located in Calabria (Figures 1 and 3) and corresponds to the so‐called “Crati‐Sibari” plain, a region recognized as a major genetic discontinuity for several vertebrate species (Canestrelli and Nascetti 2008; Canestrelli et al. 2010). Conversely, both sPCA and Bayesian clustering analysis of nuclear microsatellites indicate that the primary genetic break settled more southern and separates Sicilian populations from all other mainland groups. This mito‐nuclear discordance is not surprising, and several factors can be argued to explain this pattern (Toews and Brelsford 2012). First of all, microsatellite loci are not well suited to time and reconstruct long‐term evolutionary processes due to their higher mutation rates; thus, the observed early separation of the ancestral components could be mainly caused by recent demographic changes and isolation processes as expected in an insular context. In fact, due to this geological history, Sicily has a high level of biogeographic distinctiveness compared to mainland Italy, exemplified by several island endemics (Schmitt et al. 2021). However, although the observed divergence, there was a certain level of gene flow between the Sicilian S3 haplogroups and the mainland (Figure 4), especially in the direction from S3 to the mainland. Interestingly, two haplotypes from Podnar et al. (2005) corresponding to populations 35, 36 and 37 in Senczuk et al. (2017), located in southern Calabria and not genotyped in this work, clearly belonged to the Sicilian clade S3. According to the level of divergence of these two haplogroups, it is difficult to date back the colonization event, but land connections during the Last Glacial Maximum (Antonioli et al. 2016) might have facilitated dispersal and introgression as occurred in other temperate species (e.g., Canestrelli and Nascetti 2008). It is therefore possible that gene flow between the S3 and S1 populations occurred within Calabria. The genetic isolation of Sicily and the observed nuclear asymmetrical gene flow from Sicily to mainland (S1), but also from A3 to S1, aligns with the dorsal phenotypic traits (Gallozzi et al. 2022). Indeed, while in Sicily the predominant morphotypes are the reticulated and concolor forms (*sensu* Gallozzi et al. 2022), Calabrian populations exhibit greater phenotypic variability, displaying a mix of dorsal patterns typical of both Sicily and the mainland. This suggests that the intraspecific nuclear differentiation observed in Sicily is also reflected at the phenotypic level (as seen in other taxa, e.g., Stöck et al. 2008) and that gene flow towards Calabria may have contributed to the high polymorphism in dorsal patterning observed in this region. This may also be the case for group A2f, which shows signs of introgression from Sicily and displays high variability in dorsal pattern traits.

Concerning Sicily, a further discrepancy between mtDNA and nuclear microsatellite loci is evident. In this region, at least 10 geographically structured haplogroups are present (Senczuk et al. 2017), likely reflecting the cyclic Pleistocene fragmentation of the island, first into paleo‐islands during the Early Pleistocene, then into distinct environmental settings during the Middle and Late Pleistocene climatic oscillations (Schmitt et al. 2021). In contrast, nuclear microsatellite analyses suggest ongoing genetic exchange among Sicilian populations, with gene flow values significantly higher than those among peninsular groups (Figure 4). Species distribution modeling for the Last Glacial Maximum suggests stable or even improved environmental conditions in Sicily, promoting population expansion during glacial periods (Senczuk et al. 2017; Sherpa et al. 2024). Considering these ecological dynamics and the known female philopatry in this species (Vignoli et al. 2012) and lizards in general (Ferchaud et al. 2015), we propose that female‐biased site fidelity is the most plausible explanation for the observed mito‐nuclear discordance in Sicily. It is also possible that the microsatellite markers used may lack sufficient resolution to detect fine‐scale genetic structure. Indeed, genomic analyses suggest a divergence at the nuclear DNA level in Sicilian populations, although they lack adequate geographic coverage (Sherpa et al. 2024).

A similar condition is also observed in the Calabrian region. Indeed, while the mitochondrial DNA identified three principal deeply divergent clades (S1, S2, and A3), probably reflecting long‐term isolation due to the complex paleogeographic history which repeatedly transformed southern Calabria into a chain of islands since the Pliocene (Schmitt et al. 2021), the microsatellite data indicated a slightly homogeneous genetic unit. The nuclear make‐up is characterized by a high frequency of genetic cluster 3, which emerges consistently in all *K* values greater than two and by moderately high gene flow values (Figures 3 and 4). These findings suggest that the mitochondrial lineage boundary does not constitute a barrier to gene flow, contrary to previous assumptions based on the strict parapatry of mitochondrial lineages (Senczuk et al. 2017). Moreover, a secondary contact zone located about 100‐km north of the main mitochondrial discontinuity (separating the S and the CN lineages) was detected at the nuclear DNA. All individuals of this area, corresponding to the haplogroup A2g, present admixture components (Figure 3). According to time‐calibrated phylogeny based on both Whole Genome Sequencing (WGS) and mitochondrial DNA, the separation of the Siculo‐Calabrian and Central‐Northern lineages is very ancient and may correspond to Early Pliocene or even before (Senczuk et al. 2017; Senczuk, Havenstein, et al. 2018; Senczuk, Colangelo, et al. 2018; Yang et al. 2021). Hybridization and introgression between distant related lineages or even taxa has been shown to be a major prompt in driving the exceptional diversity and adaptation of the *Podarcis* lizards (Yang et al. 2021). The found northward shift in the position of the nuclear secondary contact compared to the mitochondrial cline mirrors the asymmetric hybridization observed in the common wall lizard *Podarcis muralis* (While et al. 2015). In that case, differences in male–male competition between diverging lineages would seem to have driven the replacement of the nuclear alleles of the subdominant lineage generating asymmetric introgression upon secondary contact. Although morphological differences at this contact zone have been already documented (Gallozzi et al. 2022), further investigation using phenotypic traits and genome‐wide data would be necessary to confirm the extent of the nuclear introgression and to identify candidate genes responsible for adaptive introgression.

In the northernmost populations belonging to the Central‐Northern lineage, the two clades, Tyrrhenian (T) and Adriatic (A), are present with a west–east distribution across the Apennine ridge (Figure 1). This pattern has been observed in other species, such as the western whip snake *Hierophis viridiflavus* (Senczuk et al. 2021), but it is not common among Italian vertebrates (Schmitt et al. 2021). The genetic clustering at nuclear markers appears to be largely consistent with that observed at mitochondrial markers (Figure 3). At *K* = 4, the Tyrrhenian (T) clade is genetically distinct from the Adriatic (A) clade, with limited gene flow between them. Previous studies comparing species distribution models for *P. siculus* under present conditions and during the Last Glacial Maximum revealed differences in past habitat suitability between the western and eastern coasts (Senczuk et al. 2017). Specifically, the analysis indicated a narrow zone of suitable habitat along the northern Tyrrhenian coast, while habitat suitability was significantly lower along the Adriatic coast. From a mitochondrial lineage perspective, this is reflected in signals of prolonged demographic stability for the Tyrrhenian clade (T), whereas the northern Adriatic clade (A2) exhibits evidence of demographic expansion, beginning approximately 70 kya. These events are reflected in the genetic diversity of clade A. Within this clade, the northern haplogroup A2c emerged as the most genetically distinct in both sPCA and clustering analyses, exhibiting minimal admixture compared to other haplogroups within lineage A. The characteristics of haplogroup A2c, marked by the predominance of genetic cluster 3, likely reflect the effects of genetic drift associated with demographic expansion and subsequent relative isolation.

To conclude, although globally we do not find evidence of isolation by distance, higher levels of gene flow were detected among geographically contiguous haplogroups. This occurs especially in southern regions where the topographic setting may have facilitated dispersal dynamics or local demographic expansion.

## 5 Conclusions

This study offers new insights into the complex phylogeographic history of *P. siculus* , emphasizing the interaction between mitochondrial and nuclear markers in shaping the species' evolutionary history. The previous findings based on mitochondrial data are confirmed, indicating that, from a nuclear perspective as well, several genetically differentiated groups exist. In many respects, this outcome was expected, as it reflects the influence of ancient Pleistocene paleoclimatic events, which led to the formation of micro‐refugia across the Italian Peninsula. Nonetheless, some differences emerged between the distribution of nuclear and mitochondrial groups. Although we are unable to definitively identify the determinants of the observed discordance, our findings point to multiple contributing factors, among which are the distinct evolutionary timescales reflected by mitochondrial DNA and microsatellite markers, different levels of introgression of different markers across hybrid zones, differences in competition for mates or distinct dispersal patterns, potentially influenced by sex‐biased philopatry. The species warrants further investigation at a finer geographic scale, focusing on contact zones and hybridization between mitochondrial lineages. A more detailed genomic scan would provide deeper insights into gene flow patterns and help identify potential reproductive isolation mechanisms. In addition, experimental designs aimed at highlighting competition between males of different lineages could provide insights into potential reproductive barriers, fitness differences, or the direction of gene flow (e.g., MacGregor et al. 2017). Finally, comparative phylogeographic analyses of species co‐distributed with *P. siculus* but exhibiting different ecological requirements (e.g., *P. waglerianus* and *P. muralis* ) could help elucidate additional factors shaping the genetic structure of populations.

## Author Contributions

**Gabriele Senczuk:** conceptualization (equal), formal analysis (equal), investigation (equal), methodology (equal), writing – original draft (lead), writing – review and editing (equal). **Chiara Ripa:** data curation (equal), formal analysis (equal), writing – original draft (equal), writing – review and editing (equal). **Paolo Colangelo:** formal analysis (equal), investigation (equal), methodology (equal), writing – original draft (equal), writing – review and editing (equal). **Riccardo Castiglia:** conceptualization (equal), funding acquisition (lead), writing – original draft (equal), writing – review and editing (equal).

## Funding

This work was supported by Consiglio Nazionale delle Ricerche and Ministero dell'Università e della Ricerca B83C22002930006.

## Conflicts of Interest

The authors declare no conflicts of interest.

## Supporting information


**Appendix S1:** ece372655‐sup‐0001‐AppendixS1.pdf.

## Data Availability

Data can be accessed at this link: https://zenodo.org/records/14948089.
