# Supplementary material for: Genetic Population Structure of the Italian Wall Lizards Podarcis siculus (Squamata: Lacertidae): Insight From Nuclear DNA Markers
Source: Ecol Evol. 2026 Jan 15;16(1):e72655. doi: 10.1002/ece3.72655 (PMC12808333; doi:10.1002/ece3.72655)

Supplementary files

Genetic population structure of the Italian wall lizards *Podarcis siculus* (Squamata: Lacertidae): insight from Nuclear DNA markers

Gabriele Senczuk[1], Chiara Ripa[2], Paolo Colangelo[3,] and Riccardo Castiglia[4]

1. Dipartimento di Agricoltura, Ambiente e Alimenti, Università del Molise, Campobasso, Italia g.senczuk@unimol.it
2. National Research Council, Institute of Polar Sciences c/o Scientific Campus - Ca' Foscari University Venice - Via Torino, 155 - 30172 Venezia Mestre (VE). chiara.ripa@cnr.it
3. National Research Council, Research Institute on Terrestrial Ecosystems, via Salaria km 29.300, 00010, Montelibretti (Rome), Italy. paolo.colangelo@cnr.it
4. Università degli studi di Roma “La Sapienza”, Dipartimento di Biologia e Biotecnologie “Charles Darwin”, via Borelli 50, 00188, Rome, Italy. riccardo.castiglia@uniroma1.it

Table A1. Individual ID, locality codes, sampling localities with geographic coordinates and the name of the haplogroups of each individual. The locality codes and haplogroups match those shown on the map in Fig. 1 and are consistent with those reported in Senczuk et al. (2017).

Individual ID	Locality code	Locality	Lat	Long	clade
1aS_273	1aS	Vulcanello	38,421	14,959	S3ieo
1aS_274	1aS	Vulcanello	38,421	14,959	S3ieo
1aS_275	1aS	Vulcanello	38,421	14,959	S3ieo
1aS_277	1aS	Vulcanello	38,421	14,959	S3ieo
1bS_278	1bS	Vulcano	38,403	14,950	S3ieo
1bS_279	1bS	Vulcano	38,403	14,950	S3ieo
1bS_280	1bS	Vulcano	38,403	14,950	S3ieo
1bS_281	1bS	Vulcano	38,403	14,950	S3ieo
1bS_282	1bS	Vulcano	38,403	14,950	S3ieo
2aS_283	2aS	Salina Malfa	38,580	14,833	S3ieo
2aS_284	2aS	Salina Malfa	38,580	14,833	S3ieo
2aS_285	2aS	Salina Malfa	38,580	14,833	S3ieo
2bS_294	2bS	Salina Pollara	38,578	14,806	S3ieo
3S_ 286	3S	Lipari	38,454	14,955	S3ieo
3S_ 287	3S	Lipari	38,454	14,955	S3ieo
4S_ 288	4S	Stromboli	38,808	15,229	S3ieo
4S_ 289	4S	Stromboli	38,808	15,229	S3ieo
4S_ 290	4S	Stromboli	38,808	15,229	S3ieo
4S_ 291	4S	Stromboli	38,808	15,229	S3ieo
4S_ 292	4S	Stromboli	38,808	15,229	S3ieo

5S_ 271	5S	Capo D’orlando	38,152	14,760	S3i
6S_ 15	6S	Nebrodi	37,874	14,626	S3i
6S_ 16	6S	Nebrodi	37,874	14,626	S3i
6S_ 266	6S	Nebrodi	37,874	14,626	S3g
6S_ 267	6S	Nebrodi	37,874	14,626	S3g
7S_ 262	7S	Capizzi	37,809	14,475	S3i
8S_ 258	8S	Pollina	38,011	14,144	S3h
8S_ 259	8S	Pollina	38,011	14,144	S3i
8S_ 260	8S	Pollina	38,011	14,144	S3i
8S_ 261	8S	Pollina	38,011	14,144	S3h
9S_ 243	9S	Roccapalumba	37,825	13,637	S3h
9S_ 244	9S	Roccapalumba	37,825	13,637	S3h
9S_254	9S	Roccapalumba	37,825	13,637	S3h
12S_257	12S	Caltavuturo	37,831	13,890	S3h
13S_94	13S	Sferrocavallo	38,210	13,288	S3h
13S_95	13S	Sferrocavallo	38,210	13,288	S3h
14S_18	14S	Zingaro	38,094	12,798	S3e
14S_22	14S	Zingaro	38,094	12,798	S3e
14S_25	14S	Zingaro	38,094	12,798	S3h
14S_48	14S	Zingaro	38,094	12,798	S3e
14S_49	14S	Zingaro	38,094	12,798	S3e
14S_51	14S	Zingaro	38,094	12,798	S3e
14S_54	14S	Zingaro	38,094	12,798	S3e
16S_56	16S	Favignana	37,915	12,320	S3heg
16S_57	16S	Favignana	37,915	12,320	S3heg
16S_59	16S	Favignana	37,915	12,320	S3heg
16S_61	16S	Favignana	37,915	12,320	S3heg
16S_91	16S	Favignana	37,915	12,320	S3heg
16S_93	16S	Favignana	37,915	12,320	S3heg
17S_71	17S	Marettimo	37,970	12,071	S3heg
17S_72	17S	Marettimo	37,970	12,071	S3heg
18S_96	18S	Stagnone	37,866	12,486	S3h
18S_97	18S	Stagnone	37,866	12,486	S3h
18S_98	18S	Stagnone	37,866	12,486	S3h
18S_99	18S	Stagnone	37,866	12,486	S3h
19S_101	19S	Rilievo Fiume	37,884	12,528	S3h
19S_102	19S	Rilievo Fiume	37,884	12,528	S3h
19S_103	19S	Rilievo Fiume	37,884	12,528	S3h
19S_104	19S	Rilievo Fiume	37,884	12,528	S3h
19S_105	19S	Rilievo Fiume	37,884	12,528	S3h
20S_128	20S	Borgo Fazio	37,854	12,714	S3h
20S_129	20S	Borgo Fazio	37,854	12,714	S3i
20S_130	20S	Borgo Fazio	37,854	12,714	S3h
21S_136	21S	Fiume Balata	37,872	12,604	S3i
21S_137	21S	Fiume Balata	37,872	12,604	S3h
21S_138	21S	Fiume Balata	37,872	12,604	S3h
22S_141	22S	Mazara	37,707	12,652	S3h
22S_142	22S	Mazara	37,707	12,652	S3h
23S_152	23S	Menfi	37,586	12,997	S3h

24S_26	24S	Eraclea Minoa	37,384	13,306	S3h
25S_153	25S	Siculiana	37,341	13,391	S3h
25S_154	25S	Siculiana	37,341	13,391	S3h
25S_157	25S	Siculiana	37,341	13,391	S3h
25S_157b	25S	Siculiana	37,341	13,391	S3h
26S_239	26S	Milena	37,482	13,710	S3g
26S_240	26S	Milena	37,482	13,710	S3g
26S_241	26S	Milena	37,482	13,710	S3g
26S_242	26S	Milena	37,482	13,710	S3g
28S_232	28S	Mazzarino	37,311	14,190	S3d
28S_233	28S	Mazzarino	37,311	14,190	S3g
28S_234	28S	Mazzarino	37,311	14,190	S3g
28S_235	28S	Mazzarino	37,311	14,190	S3g
29S_231	29S	L. Disueri	37,435	14,100	S3d
30S_160	30S	Manfria	37,105	14,157	S3g
30S_161	30S	Manfria	37,105	14,157	S3g
30S_163	30S	Manfria	37,105	14,157	S3g
31S_223	31S	Sughereta	37,071	14,439	S3d
31S_226	31S	Sughereta	37,071	14,439	S3d
31S_227	31S	Sughereta	37,071	14,439	S3d
31S_228	31S	Sughereta	37,071	14,439	S3d
31S_229	31S	Sughereta	37,071	14,439	S3d
31S_230	31S	Sughereta	37,071	14,439	S3d
32S_220	32S	Vittoria	36,968	14,550	S3d
32S_222	32S	Vittoria	36,968	14,550	S3d
34S_214	34S	Pachino	36,731	15,007	S3b
34S_215	34S	Pachino	36,731	15,007	S3b
34S_217	34S	Pachino	36,731	15,007	S3b
35S_29	35S	Porto Palo	36,716	15,121	S3i
36S_30	36S	Vendicari	36,803	15,096	S3b
36S_31	36S	Vendicari	36,803	15,096	S3b
36S_32	36S	Vendicari	36,803	15,096	S3b
36S_34	36S	Vendicari	36,803	15,096	S3b
36S_35	36S	Vendicari	36,803	15,096	S3d
36S_36	36S	Vendicari	36,803	15,096	S3b
36S_37	36S	Vendicari	36,803	15,096	S3b
36S_38	36S	Vendicari	36,803	15,096	S3b
36S_39	36S	Vendicari	36,803	15,096	S3b
36S_40	36S	Vendicari	36,803	15,096	S3b
37S_210	37S	Noto Lido	36,847	15,110	S3b
37S_211	37S	Noto Lido	36,847	15,110	S3b
37S_212	37S	Noto Lido	36,847	15,110	S3b
38S_209	38S	Cavagrande	36,985	15,030	S3b
39S_165	39S	Cassibile	36,982	15,165	S3b
39S_166	39S	Cassibile	36,982	15,165	S3b
40S_204	40S	Canicattini	37,064	15,122	S3b
40S_205	40S	Canicattini	37,064	15,122	S3b
40S_208	40S	Canicattini	37,064	15,122	S3b
41S_203	41S	Florida	37,117	15,141	S3b

42S_42	42S	Saline di Priolo	37,153	15,180	S3b
42S_44	42S	Saline di Priolo	37,153	15,180	S3b
43S_199	43S	Sorciano	37,160	15,159	S3b
44S_169	44S	Lentino	37,334	14,999	S3b
44S_170	44S	Lentino	37,334	14,999	S3i
45S_196	45S	Foce Simeto	37,378	15,035	S3f
46S_197	46S	Corridore del pero	37,321	15,045	S3b
47S_182	47S	Acireale	37,598	15,172	S3f
47S_183	47S	Acireale	37,598	15,172	S3f
47S_184	47S	Acireale	37,598	15,172	S3f
47S_185	47S	Acireale	37,598	15,172	S3f
47S_187	47S	Acireale	37,598	15,172	S3f
47S_189	47S	Acireale	37,598	15,172	S3f
48S_190	48S	Trecastagne	37,621	15,091	S3f
48S_191	48S	Trecastagne	37,621	15,091	S3f
48S_193	48S	Trecastagne	37,621	15,091	S3f
49S_194	49S	Pedara	37,617	15,048	S3f
50S_181	50S	Scura	37,664	15,155	S3f
51S_175	51S	Pozzillo	37,672	15,193	S3f
51S_176	51S	Pozzillo	37,672	15,193	S3i
51S_177	51S	Pozzillo	37,672	15,193	S3i
51S_178	51S	Pozzillo	37,672	15,193	S3i
51S_179	51S	Pozzillo	37,672	15,193	S3i
51S_180	51S	Pozzillo	37,672	15,193	S3i
52S_01	52S	Isola bella (spiag.)	37,851	15,298	S3i
52S_06	52S	Isola bella (spiag.)	37,851	15,298	S3i
52S_07	52S	Isola bella (spiag.)	37,851	15,298	S3i
53S_02	53S	Isola bella	37,850	15,300	S3i
53S_03	53S	Isola bella	37,850	15,300	S3i
53S_04	53S	Isola bella	37,850	15,300	S3i
53S_05	53S	Isola bella	37,850	15,300	S3i
54S_08	54S	Gaggi	37,861	15,210	S3i
55S_09	55S	Gole Alcantara	37,872	15,166	S3i
55S_11	55S	Gole Alcantara	37,872	15,166	S3i
55S_12	55S	Gole Alcantara	37,872	15,166	S3i
55S_13	55S	Gole Alcantara	37,872	15,166	S3i
1CL_138	1CL	Gerace	38,276	16,220	S2
1CL_140	1CL	Gerace	38,276	16,220	S2
2CL_141	2CL	Mammola	38,364	16,243	S2
2CL_142	2CL	Mammola	38,364	16,243	S2
3CL_127	3CL	Maida	38,858	16,386	S2
4CL_113	4CL	Rende	39,331	16,185	S1
4CL_114	4CL	Rende	39,331	16,185	S1
5CL_136	5CL	Mileto	38,608	16,061	S1
5CL_137	5CL	Mileto	38,608	16,061	S1
6CL_128	6CL	Vena di Maida	38,859	16,369	S1
6CL_129	6CL	Vena di Maida	38,859	16,369	S1
6CL_130	6CL	Vena di Maida	38,859	16,369	S1

6CL_131	6CL	Vena di Maida	38,859	16,369	S1
7CL_144	7CL	Simeri-Crichi foce	38,849	16,678	S1
7CL_145	7CL	Simeri-Crichi foce	38,849	16,678	S1
7CL_146	7CL	Simeri-Crichi foce	38,849	16,678	S1
7CL_147	7CL	Simeri-Crichi foce	38,849	16,678	S1
8CL_148	8CL	Steccato di Cutro	38,938	16,910	S1
8CL_149	8CL	Steccato di Cutro	38,938	16,910	S1
8CL_150	8CL	Steccato di Cutro	38,938	16,910	S1
11CL_119	11CL	Falerna	39,000	16,178	A3
12CL_151	12CL	Altilia	39,130	16,256	A3
12CL_152	12CL	Altilia	39,130	16,256	A3
12CL_153	12CL	Altilia	39,130	16,256	A3
12CL_154	12CL	Altilia	39,130	16,256	A3
13CL_115	13CL	Belmonte	39,161	16,083	A3
13CL_116	13CL	Belmonte	39,161	16,083	A3
14CL_108	14CL	Fiumefreddo	39,232	16,075	A3
14CL_109	14CL	Fiumefreddo	39,232	16,075	A3
14CL_110	14CL	Fiumefreddo	39,232	16,075	A3
14CL_111	14CL	Fiumefreddo	39,232	16,075	A3
14CL_112	14CL	Fiumefreddo	39,232	16,075	A3
16CL_101	16CL	Cirella	39,699	15,802	A2g
16CL_102	16CL	Cirella	39,699	15,802	A2g
16CL_103	16CL	Cirella	39,699	15,802	A2g
17CL_105	17CL	Scalea	39,820	15,793	A2g
18CL_106	18CL	Praia a Mare	39,889	15,786	A2g
18CL_107	18CL	Praia a Mare	39,889	15,786	A2g
20CL_156	20CL	Rossano	39,578	16,635	S1
20CL_157	20CL	Rossano	39,578	16,635	S1
20CL_158	20CL	Rossano	39,578	16,635	S1
20CL_159	20CL	Rossano	39,578	16,635	S1
20CL_160	20CL	Rossano	39,578	16,635	S1
21CL_164	21CL	Marina di Mandatorricci o	39,537	16,866	S1
21CL_167	21CL	Marina di Mandatorricci o	39,537	16,866	S1
22CL_170	22CL	Cirò marina	39,361	17,126	S1
22CL_171	22CL	Cirò marina	39,361	17,126	S1
1P_ 06	1P	Gravina	40,796	16,423	A2d
2P_ 07	2P	Rosa Marina	40,793	17,556	A2d
3P_ 08	3P	S. Domino	42,117	15,494	A2d
1C_ 14	1C	Rovine di Velia	40,162	15,164	A2f
1C_ 15	1C	Rovine di Velia	40,162	15,164	A2f
1C_ 16	1C	Rovine di Velia	40,162	15,164	A2f
1C_ 17	1C	Rovine di Velia	40,162	15,164	A2f
1C_ 18	1C	Rovine di Velia	40,162	15,164	A2f
3C_ 13	3C	Pioppi	40,234	14,945	A2f

4C_ 07	4C	Punta Licosa	40,234	14,945	A2f
4C_ 08	4C	Punta Licosa	40,234	14,945	A2f
4C_ 09	4C	Punta Licosa	40,234	14,945	A2f
4C_ 10	4C	Punta Licosa	40,234	14,945	A2f
4C_ 11	4C	Punta Licosa	40,234	14,945	A2f
4C_ 12	4C	Punta Licosa	40,234	14,945	A2f
5C_ 05	5C	Capaccio	40,417	15,095	A2f
5C_ 06	5C	Capaccio	40,417	15,095	A2f
6C_ 02	6C	Roscigno	40,414	15,335	A2f
6C_ 03	6C	Roscigno	40,414	15,335	A2f
7C_ 19	7C	R.N. Sale Tanagro	40,520	14,924	A2f
7C_ 20	7C	R.N. Sale Tanagro	40,520	14,924	A2f
7C_ 21	7C	R.N. Sale Tanagro	40,520	14,924	A2f
7C_ 22	7C	R.N. Sale Tanagro	40,520	14,924	A2d
8C_ 23	8C	Amalfi	40,636	14,682	A2f
9C_ 24	9C	Ercolano	40,799	14,352	A2d
9C_ 25	9C	Ercolano	40,799	14,352	A2d
9C_ 26	9C	Ercolano	40,799	14,352	A2b
9C_ 27	9C	Ercolano	40,799	14,352	A2d
10C_30	10C	Palma Campania	40,868	14,562	A2b
10C_31	10C	Palma Campania	40,868	14,562	A2b
10C_32	10C	Palma Campania	40,868	14,562	A2b
11C_33	11C	Liveri	40,901	14,582	A2b
12C_41	12C	Conza	40,841	15,291	A2d
12C_42	12C	Conza	40,841	15,291	A2d
13C_29	13C	Monteaguto	41,256	15,237	A2d
2L_ 46	2L	Sperlonga	41,261	13,448	Tc
2L_ 47	2L	Sperlonga	41,261	13,448	Td
2L_ 48	2L	Sperlonga	41,261	13,448	Td
3L_ 19	3L	Circeo	41,355	13,058	Td
4L_ 49	4L	Lago dei Monaci	41,405	12,876	Td
4L_ 50	4L	Lago dei Monaci	41,405	12,876	Td
4L_ 51	4L	Lago dei Monaci	41,405	12,876	Tc
4L_ 52	4L	Lago dei Monaci	41,405	12,876	Tc
4L_ 53	4L	Lago dei Monaci	41,405	12,876	Td
4L_ 20	4L	Lago dei Monaci	41,405	12,876	Tc
9L_ 23	9L	Foglino	41,473	12,716	Tc
9L_ 39	9L	Foglino	41,473	12,716	Tc
9L_ 40	9L	Foglino	41,473	12,716	Tc
9L_ 41	9L	Foglino	41,473	12,716	Tc
9L_ 43	9L	Foglino	41,473	12,716	Tc
9L_ 44	9L	Foglino	41,473	12,716	Tc
10L_32	10L	Castelporziano	41,706	12,419	Tc
10L_34	10L	Castelporziano	41,706	12,419	Tc
11L_71	11L	Anagni	41,738	13,161	Td
11L_72	11L	Anagni	41,738	13,161	Td

12L_11	12L	Bellegra	41,878	13,030	Td
12L_12	12L	Bellegra	41,878	13,030	Td
12L_13	12L	Bellegra	41,878	13,030	Td
12L_14	12L	Bellegra	41,878	13,030	Td
12L_74	12L	Bellegra	41,878	13,030	Td
13L_75	13L	Olevano Romano	41,861	13,038	Td
15L_30	15L	P. della Mola	42,156	12,149	Tc
16L_15	16L	Maccarese	41,891	12,276	Tc
18L_05	18L	M. della Tolfa	42,137	11,971	Tc
19L_18	19L	Foce Verde	41,387	12,924	Tc
1A_ 01	1A	Atri	42,580	13,979	A2d
1U_ 01	1U	E45 km117	43,319	12,386	Tb
2U_ 02	2U	E45 km93	42,989	12,375	Tb
1T_ 03	1T	Giannella	42,461	11,183	Tb
1T_ 04	1T	Giannella	42,461	11,183	Tb
1T_ 05	1T	Giannella	42,461	11,183	Tb
1T_ 06	1T	Giannella	42,461	11,183	Tb
1T_ 09	1T	Giannella	42,461	11,183	Tb
2T_ 07	2T	Feniglia	42,418	11,239	Tb
2T_ 08	2T	Feniglia	42,418	11,239	Tb
3T_01	3T	Sanrossore	43,700	10,323	Tb
1M_ 07	1M	Tavullia	43,884	12,761	A2c
1M_ 08	1M	Tavullia	43,884	12,761	A2c
1M_ 09	1M	Tavullia	43,884	12,761	A2c
1M_ 10	1M	Tavullia	43,884	12,761	A2c
1M_ 11	1M	Tavullia	43,884	12,761	A2c
2M_ 12	2M	Mondaino	43,873	12,648	A2c
3M_ 15	3M	M. Conca	43,872	12,498	A2c
3M_ 16	3M	M. Conca	43,872	12,498	A2c
3M_ 17	3M	M. Conca	43,872	12,498	A2c
1E_ 01	1E	Mesola	44,856	12,244	A2c
1E_ 02	1E	Mesola	44,856	12,244	A2c
1E_ 03	1E	Mesola	44,856	12,244	A2c
1E_ 04	1E	Mesola	44,856	12,244	A2c
1E_ 05	1E	Mesola	44,856	12,244	A2c
1E_ 06	1E	Mesola	44,856	12,244	A2c
1E_ 08	1E	Mesola	44,856	12,244	A2c

Table A2. PCR cycles. Ta: specific temperature for each locus (see Tab. A3), Tb: temperature with 3° degrees more than Ta)

	**Temperature**	**Time**	**Cicles**
DENATURATION	94°	5 min	
DENATURATION<br>ANNEALING<br>ELONGATION	94°<br>Ta<br>72°	30 sec<br>1 min<br>45 sec	5x
DENATURATION<br>ANNEALING<br>ELONGATION	94°<br>Tb<br>72°	30 sec<br>1 min<br>45 sec	35x
ELONGATION	72°	10 min	

Table A3. Characteristics of the microsatellite loci used: repeat motif, primers for amplification, and annealing temperature (*mix1; **mix 2).

**Locus**	**Repeat motif**	**Sequence of primer**	**Temp**	**Reference**
Pb73*	(CA)*n*CT(CA)*n*	GCCCATGTCACTTCAGGTAGAAGC<br>GAAAACTAGGAGTTAGGGAGAAGG	56°	Pinho et al. (2004)
Pli10*	$(CTTT)_6(CCTT)(CTTT)_4$	TGACTTGTAGGGCTGGCTTT<br>AGCTGTTTCTCAGCTGTGGTC	56°	Bloor et al. (2011)
Pli3	$(GAAA)_{12}$	CATGAAGGGAGGCGATGTAT<br>GATCCCATTCTGTCTTGGAA	58°	Bloor et al. (2011)
C9	$(CAA)_7CCA$	CATTGCTGGTTCTGGAGAAAG<br>CCTGATGAAGGGAAGTGGTG	58°	Nembini & Opplinger (2003)
Pli24**	$(CTGT)_7$	CCACAAGGACTCAGGCTCTC<br>TCCCCCACTTAAGCATGTTC	57°	Bloor et al. (2011)
Pli4**	$(CTTT)_{14}$	TCAGTTCATGCATAAGGTCCA<br>TTCGGCATTTTTCTTCAGGT	57°	Bloor et al. (2011)
Pli18	$(CTTT)_{13}$	CAAGAATTGAGTTTGCAGTTCC<br>TGTCTGACAGAATGTGCTTCTC	58°	Bloor et al. (2011)
Pb10	(GT)*n*GC(GT)*n*GC(GT)*n*(AG)*n*	AGTGGAATCGGCTGCAATAC<br>ACCAGTCCCAGGAATTTAGG	58°	Pinho et al. (2004)
Pli21	$(CTTT)_{12}$	CCATTATGACCTTGCTGGTG<br>GAACTCTGGTGGCCCACAT	58°	Bloor et al. (2011)
Lv19	$(AC)_{22}$	CTGTTGCTATTTTGTATGCTTAC<br>CCTGTGACTGTCCTCAGAGG	57°	Boudjemadi et al. (1999)
Lv4a	$(AC)_{14}A(GA)_{11}(CAGAGA)_9CAGAT(AG)_3$	CTGCAGGGAACAGAATTAACC<br>CTGCCCAGAAAGCATTTCC	60°	Boudjemadi et al. (1999)

Table A4: Frequency of null alleles for the 11 microsatellite loci in each group.

Population	Locus	Observed	Median.boot	2.5%	97.5%
A2b	Locus1	0.320	0.320	0.000	0.480
A2b	Locus2	-0.070	-0.100	-0.170	-0.070
A2b	Locus3	-0.038	-0.057	-0.210	0.117
A2b	Locus4	-0.043	-0.050	-0.178	0.000
A2b	Locus5	0.320	0.320	0.000	0.480
A2b	Locus6	0.044	0.022	-0.130	0.143
A2b	Locus7	0.033	0.000	-0.141	0.143
A2b	Locus8	0.450	0.320	0.038	0.640
A2b	Locus9	0.320	0.320	0.000	0.480
A2b	Locus10	0.117	0.114	0.000	0.117
A2b	Locus11	-0.089	-0.113	-0.250	0.038
Tc	Locus1	0.171	0.147	0.024	0.323
Tc	Locus2	-0.003	-0.017	-0.057	0.039
Tc	Locus3	0.019	0.000	-0.087	0.131
Tc	Locus4	-0.001	-0.014	-0.087	0.091
Tc	Locus5	0.091	0.074	-0.038	0.243
Tc	Locus6	0.147	0.128	-0.023	0.311
Tc	Locus7	0.125	0.107	-0.009	0.255
Tc	Locus8	0.411	0.374	0.185	0.625
Tc	Locus9	0.231	0.212	0.000	0.447
Tc	Locus10	0.095	0.077	-0.031	0.236
Tc	Locus11	0.165	0.140	-0.006	0.350
A3	Locus1	0.067	0.067	0.000	0.125
A3	Locus2	0.021	0.000	-0.090	0.121
A3	Locus3	0.054	0.032	-0.083	0.190
A3	Locus4	0.188	0.164	-0.002	0.369
A3	Locus5	-0.019	-0.019	-0.072	0.000
A3	Locus6	-0.042	-0.057	-0.087	-0.043
A3	Locus7	0.182	0.149	-0.004	0.362
A3	Locus8	0.181	0.157	-0.030	0.431
A3	Locus9	0.182	0.159	-0.031	0.411
A3	Locus10	0.204	0.181	-0.039	0.463
A3	Locus11	-0.116	-0.123	-0.188	-0.049
Td	Locus1	0.369	0.357	0.098	0.582
Td	Locus2	-0.017	-0.034	-0.094	0.047
Td	Locus3	0.033	0.014	-0.104	0.189
Td	Locus4	0.106	0.079	-0.077	0.308
Td	Locus5	-0.095	-0.098	-0.163	-0.045
Td	Locus6	0.025	0.005	-0.075	0.124
Td	Locus7	0.200	0.170	0.022	0.373
Td	Locus8	0.061	0.034	-0.076	0.194
Td	Locus9	0.095	0.080	-0.076	0.305
Td	Locus10	0.126	0.109	-0.003	0.252
Td	Locus11	0.056	0.039	-0.113	0.252
A2d	Locus1	0.404	0.354	0.115	0.587
A2d	Locus2	-0.011	-0.030	-0.101	0.071
A2d	Locus3	0.035	0.002	-0.113	0.171
A2d	Locus4	0.014	-0.010	-0.107	0.099

A2d	Locus5	0.112	0.112	-0.048	0.337
A2d	Locus6	0.002	-0.017	-0.085	0.067
A2d	Locus7	0.096	0.067	-0.054	0.241
A2d	Locus8	0.284	0.253	0.033	0.524
A2d	Locus9	0.280	0.264	-0.014	0.530
A2d	Locus10	0.218	0.190	0.071	0.371
A2d	Locus11	0.029	0.003	-0.110	0.202
S3h	Locus1	0.064	0.059	0.000	0.110
S3h	Locus2	0.041	0.030	-0.036	0.112
S3h	Locus3	-0.085	-0.092	-0.133	-0.042
S3h	Locus4	0.025	0.015	-0.054	0.111
S3h	Locus5	-0.007	-0.010	-0.053	0.037
S3h	Locus6	0.069	0.060	-0.011	0.151
S3h	Locus7	0.203	0.191	0.076	0.320
S3h	Locus8	0.087	0.076	-0.016	0.195
S3h	Locus9	0.230	0.226	0.061	0.398
S3h	Locus10	0.323	0.307	0.181	0.474
S3h	Locus11	-0.007	-0.013	-0.109	0.113
S3e	Locus1	-0.012	-0.012	-0.083	0.000
S3e	Locus2	-0.076	-0.098	-0.190	0.034
S3e	Locus3	0.042	0.008	-0.153	0.229
S3e	Locus4	0.052	0.000	-0.083	0.125
S3e	Locus5	-0.012	-0.012	-0.083	0.000
S3e	Locus6	-0.076	-0.097	-0.181	-0.076
S3e	Locus7	-0.083	-0.111	-0.174	-0.083
S3e	Locus8	0.074	0.046	-0.111	0.146
S3e	Locus9	0.393	0.345	-0.042	0.667
S3e	Locus10	0.278	0.278	0.000	0.500
S3e	Locus11	-0.133	-0.133	-0.250	-0.042
A2g	Locus1	-0.042	-0.042	-0.133	0.000
A2g	Locus2	-0.091	-0.114	-0.189	0.000
A2g	Locus3	-0.045	-0.068	-0.194	0.065
A2g	Locus4	-0.098	-0.114	-0.215	0.009
A2g	Locus5	0.000	0.000	0.000	0.000
A2g	Locus6	-0.056	-0.083	-0.139	-0.063
A2g	Locus7	-0.083	-0.111	-0.160	-0.083
A2g	Locus8	0.139	0.102	-0.189	0.440
A2g	Locus9	0.000	0.000	0.000	0.000
A2g	Locus10	0.125	0.107	-0.114	0.125
A2g	Locus11	-0.033	-0.033	-0.250	0.208
S3heg	Locus1	0.206	0.165	-0.063	0.469
S3heg	Locus2	-0.105	-0.121	-0.160	-0.098
S3heg	Locus3	0.067	0.043	-0.121	0.261
S3heg	Locus4	0.109	0.071	-0.121	0.382
S3heg	Locus5	0.000	0.000	0.000	0.000
S3heg	Locus6	0.063	0.031	-0.102	0.203
S3heg	Locus7	-0.059	-0.078	-0.121	-0.059
S3heg	Locus8	0.043	0.021	-0.204	0.282
S3heg	Locus9	-0.052	-0.057	-0.133	-0.007
S3heg	Locus10	0.326	0.299	-0.025	0.594
S3heg	Locus11	-0.030	-0.054	-0.188	0.156
S3ieo	Locus1	0.100	0.088	0.022	0.138

S3ieo	Locus2	0.012	-0.002	-0.066	0.086
S3ieo	Locus3	0.056	0.044	-0.050	0.170
S3ieo	Locus4	0.155	0.138	-0.003	0.311
S3ieo	Locus5	0.042	0.027	-0.058	0.171
S3ieo	Locus6	0.067	0.051	-0.036	0.162
S3ieo	Locus7	0.115	0.100	0.001	0.231
S3ieo	Locus8	0.087	0.074	-0.038	0.222
S3ieo	Locus9	0.350	0.345	0.095	0.585
S3ieo	Locus10	0.472	0.453	0.253	0.655
S3ieo	Locus11	0.063	0.051	-0.082	0.224
S2	Locus1	-0.043	-0.043	-0.144	0.000
S2	Locus2	0.100	0.057	-0.170	0.317
S2	Locus3	0.063	0.038	-0.170	0.317
S2	Locus4	-0.044	-0.078	-0.190	0.063
S2	Locus5	0.100	0.040	-0.144	0.480
S2	Locus6	-0.125	-0.140	-0.250	-0.109
S2	Locus7	0.286	0.229	-0.089	0.640
S2	Locus8	0.036	0.000	-0.250	0.125
S2	Locus9	0.000	0.000	0.000	0.000
S2	Locus10	0.560	0.480	0.000	0.640
S2	Locus11	-0.170	-0.170	-0.250	-0.130
A2f	Locus1	0.281	0.266	0.123	0.433
A2f	Locus2	0.060	0.046	-0.037	0.148
A2f	Locus3	0.214	0.194	0.050	0.382
A2f	Locus4	0.046	0.033	-0.049	0.141
A2f	Locus5	0.090	0.075	-0.035	0.212
A2f	Locus6	0.010	-0.004	-0.060	0.082
A2f	Locus7	0.085	0.067	-0.019	0.177
A2f	Locus8	-0.013	-0.027	-0.103	0.084
A2f	Locus9	0.335	0.321	0.144	0.505
A2f	Locus10	0.148	0.132	0.009	0.279
A2f	Locus11	0.046	0.030	-0.059	0.165
A2c	Locus1	0.092	0.090	-0.020	0.274
A2c	Locus2	0.111	0.094	-0.045	0.267
A2c	Locus3	-0.010	-0.026	-0.110	0.088
A2c	Locus4	-0.061	-0.077	-0.120	-0.015
A2c	Locus5	0.043	0.036	-0.074	0.212
A2c	Locus6	-0.006	-0.021	-0.069	0.053
A2c	Locus7	0.054	0.036	-0.054	0.155
A2c	Locus8	0.321	0.301	0.096	0.573
A2c	Locus9	0.227	0.219	0.000	0.477
A2c	Locus10	0.507	0.475	0.250	0.707
A2c	Locus11	-0.010	-0.026	-0.121	0.099
Tb	Locus1	0.150	0.150	-0.035	0.420
Tb	Locus2	0.097	0.062	-0.074	0.271
Tb	Locus3	0.004	-0.021	-0.141	0.176
Tb	Locus4	-0.003	-0.022	-0.148	0.150
Tb	Locus5	-0.046	-0.046	-0.126	-0.005
Tb	Locus6	-0.008	-0.026	-0.100	0.071
Tb	Locus7	0.100	0.068	-0.070	0.264
Tb	Locus8	0.071	0.047	-0.080	0.190
Tb	Locus9	0.180	0.180	0.000	0.420

Tb	Locus10	0.507	0.441	0.114	0.719
Tb	Locus11	-0.114	-0.126	-0.192	-0.028
S1	Locus1	0.107	0.096	0.010	0.209
S1	Locus2	0.199	0.182	0.066	0.336
S1	Locus3	0.116	0.106	-0.003	0.238
S1	Locus4	0.068	0.053	-0.029	0.166
S1	Locus5	0.112	0.098	-0.032	0.267
S1	Locus6	0.164	0.150	0.042	0.308
S1	Locus7	0.048	0.034	-0.036	0.138
S1	Locus8	0.106	0.092	-0.024	0.247
S1	Locus9	0.353	0.347	0.121	0.531
S1	Locus10	0.064	0.046	-0.034	0.159
S1	Locus11	0.072	0.056	-0.045	0.204
S3i	Locus1	0.300	0.280	0.147	0.453
S3i	Locus2	0.050	0.042	-0.021	0.125
S3i	Locus3	0.377	0.364	0.193	0.563
S3i	Locus4	0.216	0.201	0.079	0.361
S3i	Locus5	0.028	0.017	-0.072	0.124
S3i	Locus6	0.201	0.185	0.073	0.327
S3i	Locus7	0.034	0.025	-0.036	0.096
S3i	Locus8	0.189	0.177	0.062	0.320
S3i	Locus9	0.181	0.170	0.026	0.355
S3i	Locus10	0.153	0.142	0.030	0.273
S3i	Locus11	0.288	0.273	0.130	0.449
S3g	Locus1	0.089	0.073	-0.012	0.133
S3g	Locus2	0.188	0.162	-0.006	0.358
S3g	Locus3	0.048	0.024	-0.093	0.182
S3g	Locus4	-0.062	-0.076	-0.141	-0.006
S3g	Locus5	-0.019	-0.019	-0.065	0.000
S3g	Locus6	0.144	0.116	-0.029	0.314
S3g	Locus7	0.294	0.262	0.069	0.515
S3g	Locus8	0.081	0.055	-0.076	0.238
S3g	Locus9	0.267	0.240	0.029	0.521
S3g	Locus10	0.541	0.490	0.208	0.736
S3g	Locus11	0.056	0.031	-0.131	0.292
S3d	Locus1	0.045	0.029	-0.091	0.190
S3d	Locus2	0.067	0.043	-0.078	0.191
S3d	Locus3	-0.063	-0.084	-0.137	0.003
S3d	Locus4	0.065	0.048	-0.102	0.230
S3d	Locus5	-0.029	-0.029	-0.096	0.000
S3d	Locus6	0.066	0.035	-0.088	0.205
S3d	Locus7	0.043	0.022	-0.083	0.166
S3d	Locus8	0.307	0.264	0.043	0.559
S3d	Locus9	0.165	0.165	0.000	0.397
S3d	Locus10	0.363	0.314	0.117	0.593
S3d	Locus11	0.116	0.088	-0.082	0.315
S3b	Locus1	0.135	0.122	0.021	0.243
S3b	Locus2	0.053	0.043	-0.021	0.137
S3b	Locus3	-0.007	-0.017	-0.089	0.070
S3b	Locus4	0.115	0.101	0.004	0.226
S3b	Locus5	-0.046	-0.047	-0.084	-0.017
S3b	Locus6	0.101	0.085	-0.011	0.219

S3b	Locus7	0.113	0.100	0.011	0.222
S3b	Locus8	0.285	0.270	0.134	0.432
S3b	Locus9	0.071	0.071	0.000	0.198
S3b	Locus10	0.093	0.081	-0.017	0.197
S3b	Locus11	0.477	0.460	0.292	0.657
S3f	Locus1	0.452	0.433	0.149	0.722
S3f	Locus2	0.154	0.125	-0.009	0.302
S3f	Locus3	0.257	0.200	-0.066	0.590
S3f	Locus4	-0.002	-0.017	-0.104	0.101
S3f	Locus5	0.175	0.156	-0.012	0.356
S3f	Locus6	0.121	0.097	-0.034	0.267
S3f	Locus7	0.135	0.113	-0.042	0.302
S3f	Locus8	0.258	0.230	0.009	0.511
S3f	Locus9	0.206	0.188	0.007	0.382
S3f	Locus10	0.077	0.054	-0.061	0.213
S3f	Locus11	-0.035	-0.054	-0.165	0.115

Table A5: Estimates of deviations from Hardy-Weinberg, for each group and locus. FDR= False Discovery Rate adjusted p-value

Population	Locus	p.value	FDR
A2b	Locus1	0.112	0.193
A2b	Locus2	1.000	1.000
A2b	Locus3	0.297	0.404
A2b	Locus4	1.000	1.000
A2b	Locus5	0.107	0.186
A2b	Locus6	0.113	0.193
A2b	Locus7	0.249	0.352
A2b	Locus8	0.003	0.012
A2b	Locus9	0.116	0.195
A2b	Locus10	0.104	0.183
A2b	Locus11	0.235	0.336
Tc	Locus1	0.000	0.000
Tc	Locus2	0.660	0.806
Tc	Locus3	0.583	0.732
Tc	Locus4	0.499	0.635
Tc	Locus5	0.134	0.222
Tc	Locus6	0.012	0.036
Tc	Locus7	0.004	0.015
Tc	Locus8	0.000	0.000
Tc	Locus9	0.001	0.004
Tc	Locus10	0.107	0.186
Tc	Locus11	0.000	0.000
A3	Locus1	0.049	0.105
A3	Locus2	0.033	0.080
A3	Locus3	0.670	0.807
A3	Locus4	0.015	0.042
A3	Locus5	1.000	1.000
A3	Locus6	1.000	1.000
A3	Locus7	0.007	0.024
A3	Locus8	0.154	0.241
A3	Locus9	0.026	0.067
A3	Locus10	0.051	0.107
A3	Locus11	0.667	0.807
Td	Locus1	0.000	0.000
Td	Locus2	0.209	0.307
Td	Locus3	0.058	0.118
Td	Locus4	0.039	0.090
Td	Locus5	0.826	0.941
Td	Locus6	0.464	0.601
Td	Locus7	0.000	0.000
Td	Locus8	0.103	0.183
Td	Locus9	0.103	0.183
Td	Locus10	0.014	0.041
Td	Locus11	0.017	0.047
A2d	Locus1	0.000	0.000
A2d	Locus2	0.758	0.877
A2d	Locus3	0.060	0.120
A2d	Locus4	0.148	0.233

A2d	Locus5	0.101	0.183
A2d	Locus6	0.498	0.635
A2d	Locus7	0.087	0.162
A2d	Locus8	0.001	0.004
A2d	Locus9	0.009	0.030
A2d	Locus10	0.000	0.000
A2d	Locus11	0.320	0.432
S3h	Locus1	0.008	0.028
S3h	Locus2	0.010	0.032
S3h	Locus3	0.157	0.243
S3h	Locus4	0.009	0.030
S3h	Locus5	0.094	0.173
S3h	Locus6	0.034	0.081
S3h	Locus7	0.000	0.000
S3h	Locus8	0.176	0.269
S3h	Locus9	0.000	0.000
S3h	Locus10	0.000	0.000
S3h	Locus11	0.000	0.000
S3e	Locus1	1.000	1.000
S3e	Locus2	1.000	1.000
S3e	Locus3	0.785	0.904
S3e	Locus4	0.264	0.370
S3e	Locus5	1.000	1.000
S3e	Locus6	0.203	0.304
S3e	Locus7	1.000	1.000
S3e	Locus8	0.136	0.222
S3e	Locus9	0.040	0.092
S3e	Locus10	0.080	0.153
S3e	Locus11	0.519	0.656
A2g	Locus1	1.000	1.000
A2g	Locus2	1.000	1.000
A2g	Locus3	0.699	0.835
A2g	Locus4	0.451	0.588
A2g	Locus5	1.000	1.000
A2g	Locus6	1.000	1.000
A2g	Locus7	1.000	1.000
A2g	Locus8	0.020	0.052
A2g	Locus9	1.000	1.000
A2g	Locus10	0.018	0.049
A2g	Locus11	0.082	0.156
S3heg	Locus1	0.089	0.165
S3heg	Locus2	0.822	0.941
S3heg	Locus3	0.482	0.621
S3heg	Locus4	0.213	0.310
S3heg	Locus5	1.000	1.000
S3heg	Locus6	0.268	0.374
S3heg	Locus7	1.000	1.000
S3heg	Locus8	0.054	0.112
S3heg	Locus9	1.000	1.000
S3heg	Locus10	0.004	0.015
S3heg	Locus11	0.704	0.837
S3ieo	Locus1	0.000	0.000

S3ieo	Locus2	0.131	0.219
S3ieo	Locus3	0.239	0.340
S3ieo	Locus4	0.001	0.004
S3ieo	Locus5	0.224	0.322
S3ieo	Locus6	0.055	0.113
S3ieo	Locus7	0.000	0.000
S3ieo	Locus8	0.161	0.248
S3ieo	Locus9	0.000	0.000
S3ieo	Locus10	0.000	0.000
S3ieo	Locus11	0.035	0.082
S2	Locus1	1.000	1.000
S2	Locus2	0.376	0.505
S2	Locus3	0.625	0.776
S2	Locus4	1.000	1.000
S2	Locus5	0.624	0.776
S2	Locus6	0.659	0.806
S2	Locus7	0.010	0.032
S2	Locus8	0.292	0.399
S2	Locus9	1.000	1.000
S2	Locus10	0.013	0.038
S2	Locus11	0.043	0.098
A2f	Locus1	0.000	0.000
A2f	Locus2	0.000	0.000
A2f	Locus3	0.003	0.012
A2f	Locus4	0.032	0.079
A2f	Locus5	0.018	0.049
A2f	Locus6	0.745	0.872
A2f	Locus7	0.001	0.004
A2f	Locus8	0.209	0.307
A2f	Locus9	0.000	0.000
A2f	Locus10	0.000	0.000
A2f	Locus11	0.004	0.015
A2c	Locus1	0.066	0.130
A2c	Locus2	0.012	0.036
A2c	Locus3	0.672	0.807
A2c	Locus4	0.431	0.566
A2c	Locus5	0.083	0.156
A2c	Locus6	0.730	0.859
A2c	Locus7	0.003	0.012
A2c	Locus8	0.001	0.004
A2c	Locus9	0.001	0.004
A2c	Locus10	0.000	0.000
A2c	Locus11	0.020	0.052
Tb	Locus1	0.049	0.105
Tb	Locus2	0.141	0.223
Tb	Locus3	0.952	1.000
Tb	Locus4	0.430	0.566
Tb	Locus5	1.000	1.000
Tb	Locus6	0.709	0.838
Tb	Locus7	0.137	0.222
Tb	Locus8	0.207	0.307
Tb	Locus9	0.045	0.100

Tb	Locus10	0.000	0.000
Tb	Locus11	1.000	1.000
S1	Locus1	0.015	0.042
S1	Locus2	0.000	0.000
S1	Locus3	0.000	0.000
S1	Locus4	0.000	0.000
S1	Locus5	0.045	0.100
S1	Locus6	0.000	0.000
S1	Locus7	0.071	0.137
S1	Locus8	0.102	0.183
S1	Locus9	0.000	0.000
S1	Locus10	0.138	0.222
S1	Locus11	0.003	0.012
S3i	Locus1	0.000	0.000
S3i	Locus2	0.002	0.009
S3i	Locus3	0.000	0.000
S3i	Locus4	0.000	0.000
S3i	Locus5	0.116	0.195
S3i	Locus6	0.000	0.000
S3i	Locus7	0.214	0.310
S3i	Locus8	0.000	0.000
S3i	Locus9	0.000	0.000
S3i	Locus10	0.000	0.000
S3i	Locus11	0.000	0.000
S3g	Locus1	0.029	0.074
S3g	Locus2	0.000	0.000
S3g	Locus3	0.432	0.566
S3g	Locus4	0.140	0.223
S3g	Locus5	1.000	1.000
S3g	Locus6	0.013	0.038
S3g	Locus7	0.000	0.000
S3g	Locus8	0.179	0.272
S3g	Locus9	0.006	0.022
S3g	Locus10	0.000	0.000
S3g	Locus11	0.285	0.392
S3d	Locus1	0.192	0.290
S3d	Locus2	0.060	0.120
S3d	Locus3	0.861	0.976
S3d	Locus4	0.069	0.134
S3d	Locus5	1.000	1.000
S3d	Locus6	0.136	0.222
S3d	Locus7	0.285	0.392
S3d	Locus8	0.001	0.004
S3d	Locus9	0.035	0.082
S3d	Locus10	0.000	0.000
S3d	Locus11	0.003	0.012
S3b	Locus1	0.011	0.034
S3b	Locus2	0.030	0.075
S3b	Locus3	0.430	0.566
S3b	Locus4	0.011	0.034
S3b	Locus5	1.000	1.000
S3b	Locus6	0.007	0.024

S3b	Locus7	0.000	0.000
S3b	Locus8	0.000	0.000
S3b	Locus9	0.011	0.034
S3b	Locus10	0.046	0.101
S3b	Locus11	0.000	0.000
S3f	Locus1	0.000	0.000
S3f	Locus2	0.000	0.000
S3f	Locus3	0.030	0.075
S3f	Locus4	0.757	0.877
S3f	Locus5	0.047	0.102
S3f	Locus6	0.020	0.052
S3f	Locus7	0.033	0.080
S3f	Locus8	0.007	0.024
S3f	Locus9	0.051	0.107
S3f	Locus10	0.061	0.121
S3f	Locus11	0.657	0.806

Figure A1. Cross-validation score from tess3r, plotted against the number of ancestral populations (K from 2 to 20) of *Podarcis siculus*. The best K is found where the cross-validation score starts to plateau at the bottom.

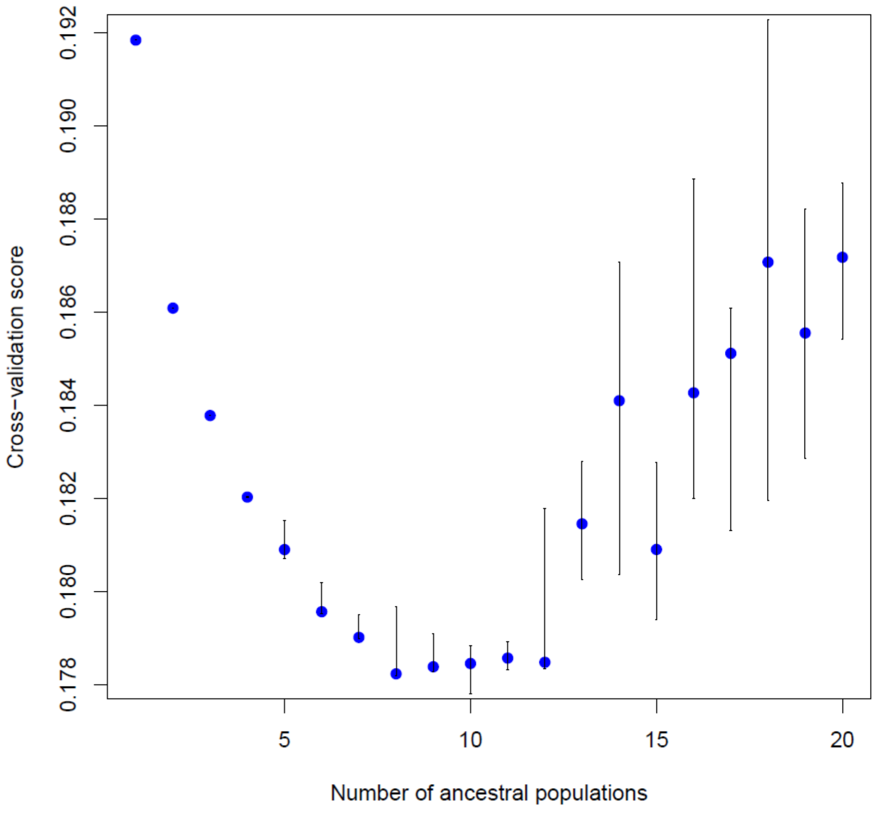

Figure A2. Relationship between genetic and geographic distances among populations of *P. siculus* based on the Mantel test. The plot shows pairwise FST values plotted against the log-transformed geographic distances (km). Although a weak positive trend is visible, the correlation was not statistically significant (p = 0.172), indicating no clear isolation-by-distance pattern among populations.

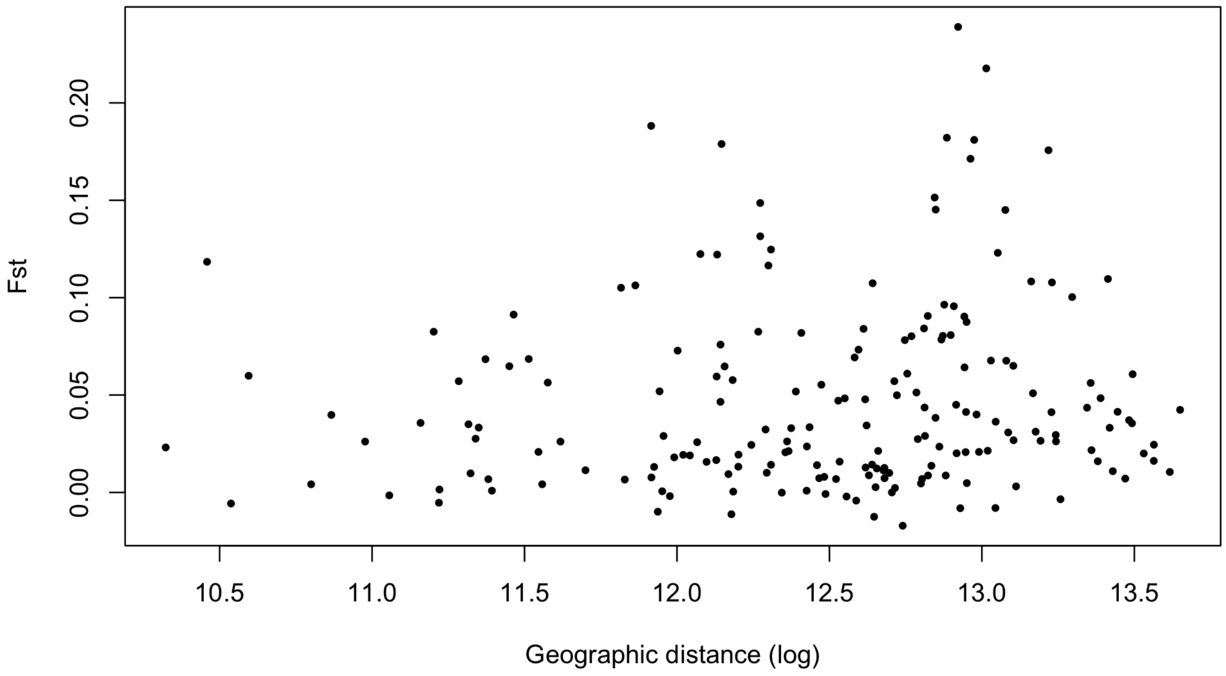

Supplement: Supplementary file 1 — Appendix S1: ece372655‐sup‐0001‐AppendixS1.pdf. [file ECE3-16-e72655-s001.pdf]
